# Factors Predicting Ictal Quality in Bilateral Electroconvulsive Therapy Sessions

**DOI:** 10.3390/brainsci11060781

**Published:** 2021-06-12

**Authors:** Aida de Arriba-Arnau, Antonia Dalmau Llitjos, Virginia Soria, Stelania Savino, Neus Salvat-Pujol, Jordi Curto, José Manuel Menchón, Mikel Urretavizcaya

**Affiliations:** 1 Neurosciences Group—Psychiatry and Mental Health, Institut d’Investigació Biomèdica de Bellvitge (IDIBELL), Department of Psychiatry, Hospital Universitari de Bellvitge-ICS, 08907 Barcelona, Spain; adearriba@bellvitgehospital.cat (A.d.A.-A.); vsoria@bellvitgehospital.cat (V.S.); nsalvat@bellvitgehospital.cat (N.S.-P.); jmenchon@bellvitgehospital.cat (J.M.M.); 2 Centro de Investigación Biomédica en Red de Salud Mental (CIBERSAM), Carlos III Health Institute, 28029 Madrid, Spain; 3 Department of Anesthesiology, Reanimation and Pain Clinic, Bellvitge University Hospital-ICS, L’Hospitalet de Llobregat, 08907 Barcelona, Spain; madalmau@bellvitgehospital.cat (A.D.L.); ssavino@bellvitgehospital.cat (S.S.); 4 Department of Clinical Sciences, School of Medicine, Faculty of Medicine and Health Sciences, Campus Bellvitge, Universitat de Barcelona (UB), 08907 Barcelona, Spain; 5 Department of Mental Health, Corporació Sanitària Parc Taulí, 08208 Sabadell, Spain; 6 Department of Public Health, Mental Health and Perinatal Nursing, School of Nursing, Faculty of Medicine and Health Sciences, Campus Bellvitge, Universitat de Barcelona (UB), 08907 Barcelona, Spain; jjcurto@ub.edu

**Keywords:** electroconvulsive therapy (ECT), electroencephalography, seizure quality, ictal adequacy, postictal suppression, hyperventilation, oxygen, treatment outcome, regression analysis, bilateral ECT

## Abstract

In electroconvulsive therapy (ECT), ictal characteristics predict treatment response and can be modified by changes in seizure threshold and in the ECT technique. We aimed to study the impact of ECT procedure-related variables that interact during each session and might influence the seizure results. Two hundred and fifty sessions of bilateral ECT in forty-seven subjects were included. Seizure results were evaluated by two different scales of combined ictal EEG parameters (seizure quality index (SQI) and seizure adequacy markers sum (SAMS) scores) and postictal suppression rating. Repeated measurement regression analyses were performed to identify predictors of each session’s three outcome variables. Univariate models identified age, physical status, hyperventilation, basal oxygen saturation, days between sessions, benzodiazepines, lithium, and tricyclic antidepressants as predictors of seizure quality. Days elapsed between sessions, higher oxygen saturation and protocolized hyperventilation application were significant predictors of better seizure quality in both scales used in multivariate models. Additionally, lower ASA classification influenced SQI scores as well as benzodiazepine use and lithium daily doses were predictors of SAMS scores. Higher muscle relaxant doses and lower applied stimulus intensities significantly influenced the postictal suppression rating. The study found several modifiable procedural factors that impacted the obtained seizure characteristics; they could be adjusted to optimize ECT session results.

## 1. Introduction

Electroconvulsive therapy (ECT) is a high-value treatment for psychiatric illnesses that provides great response rates [1,2,3] in a quick manner [4] and improves patient health-related quality of life [5].

An effective acute ECT treatment course consists of multiple, usually 6 to 12, adequate sessions [6]. The adequacy of the generalized seizure induced in ECT sessions was initially linked to the seizure duration, which was associated with therapeutic and cognitive outcomes [7]. However, the duration alone is not considered a reliable predictor of seizure adequacy and outcomes [8,9]. ECT has proven to be equally clinically effective when using antiepileptic drugs [10], benzodiazepines [11,12], or propofol [13], which shorten seizure length. On the other hand, longer seizures do not directly influence the need for fewer ECT treatments. Moreover, it is known that seizure length increases when stimulus energy applied is too close to the seizure threshold, although it is a low-quality seizure [14], and right unilateral ECT can produce seizures with poor therapeutic potency despite being of sufficient ictal duration if the dosage is not adequately suprathreshold [15,16]. Thus, there is a growing interest in finding seizure quality markers other than ictal duration [8,9] that can be evaluable in each ECT treatment session and that are tied to clinical improvements [17] to assure session adequacy, to guide clinicians’ decisions along the ECT course, and to optimize the sessions. Currently, seizure characteristics related to the amplitude and shape of the ictal electroencephalogram (EEG) are used along with the duration [6].

Several applications regarding EEG characteristics that could help apply individualized ECT treatment routinely have been proposed by Mayur et al. [17].

First, EEG characteristics can predict the response to ECT [17]. Postictal suppression [18,19], ictal amplitude measures [8,20], and seizure quality scales correlated with symptomatology improvements [21]. Some seizure quality scales combining different indexes were also useful as predictors of ECT response [22,23].

Second, changes in EEG across ECT sessions in the same patient indicate seizure threshold changes [17], given that seizure threshold can increase as treatment progresses [24,25]. Dose increases could be guided by these ictal changes [6,17] to maintain adequate suprathreshold dosing, which is important because it affects clinical outcomes [26].

Threshold increases over a treatment course were detected by percentage change in midictal amplitude and potentially by postictal suppression [27]. In addition, a stimulation strategy (Clinical and Seizure Based Stimulation) based on seizure quality instead of seizure threshold that adapts the stimulus intensity according to the clinical status of the patient and the quality of the prior seizure has been successfully used [28].

Third, EEG could discriminate between electrode positions and different stimulus doses [17,29]. EEG can also reflect and measure the impact of changes applied in the ECT technique, such as anesthetics [30], ventilation parameters [31], the time interval from the beginning of anesthesia administration until ECT stimulus application (ASTI) [32], or bispectral index (BIS) [33].

Last, it may be useful to incorporate EEG characteristics into clinical ECT algorithms [17]. As mentioned before, a stimulation strategy protocol based on seizure quality also showed good results with brief and ultrabrief pulses [28]. Additionally, seizure quality scale scores at an early stage of the treatment course (second ECT session [34] or fourth and sixth sessions [22]) predicted treatment response and would allow us to promptly apply changes in the technique to enhance outcomes [34].

Recent research has looked for modifiable factors related to seizure characteristics and found that ECT number and the frequency of the stimulus applied were predictors of ictal duration, and that pulse width, ketamine use, stimulus duration, number of ECT sessions, percentage of energy, and frequency were predictors of postictal suppression (PSI) [7]. PSI was also superior when BIS was used to determine the moment of seizure induction [35], and longer ASTI was associated with better seizure quality [36].

Controversies related to ictal characteristics have arisen in the previous literature that studied the implications of several EEG characteristics and found that they had limited potential as markers of treatment adequacy [29]. However, when taking some of the single EEG quality characteristics combined in a build-up scale as an aggregate of several of these characteristics, they became an easy-to-use measure that has been linked to outcomes such as ECT response [22,23,34].

With the modifications applied to modern ECT procedures, there are several variables that might influence treatment session results. To date, there is a lack of research exploring the relationship between all the ECT factors that interact during ECT sessions and the seizure quality scales as aggregates of EEG characteristics. Thus, it is unclear which factors have more importance and thus could be modified to optimize seizure quality.

This study aimed to investigate which variables of the ECT session procedure impact the session’s seizure quality as measured by previously used scales of combined ictal EEG automated estimation parameters provided by the ECT device and manual ratings of PSI.

## 2. Materials and Methods

### 2.1. Sample

Patients were consecutively recruited through the ECT Unit at the Psychiatry Department of Bellvitge University Hospital (Hospitalet de Llobregat, Barcelona) for 6 months.

The practice of ECT was applied according to the APA Task Force on ECT [6] and the Spanish Consensus on ECT [37,38]. Written informed consent was obtained from the patients themselves or from a parent and/or legal guardian. The research protocol was approved by the Bellvitge University Hospital Clinical Research Ethics Committee, and all procedures were carried out according to the Declaration of Helsinki.

The inclusion criteria for the study were patients ≥18 years of age referred to ECT by their treating psychiatrist, receiving thiopental or propofol alone as anesthetics (without adjunct anesthetic regimens such as flumazenil, ketamine, remifentanil, or vasoactive drugs), and without past or current major neurological illness or injury. The initial sessions of the treatment courses were excluded for the analysis because multiple stimulations were performed to find the patient’s seizure threshold. Concomitant psychotropic medications were not withdrawn, but benzodiazepines were maintained at the lowest dose tolerated during the treatment course.

### 2.2. ECT Protocol

The ECT was administered using a Thymatron System IV device (Somatics, Inc., Lake Bluff, IL, USA) and brief-pulse bitemporal electrode placement in all patients. The patient’s seizure threshold was determined in the first session following the unit’s titration method protocol. In the following sessions, the stimulus was administered at 1.5–2.5 times above the patient’s seizure threshold and individually adjusted during the treatment course according to the patient’s clinical evolution and seizure quality.

The length and characteristics of the seizure were determined by 2-channel electroencephalogram (EEG) with electrodes at the Fp1 and Fp2 sites. Motor seizure length was also recorded. Anesthesia was induced with intravenous thiopental (1.5–2.5 mg/kg) or propofol (1–2.5 mg/kg), and succinylcholine (0.5–1 mg/kg) was used for muscle relaxation. During treatment sessions, blood pressure, heart rate, and ventilation gases (oxygen and carbon dioxide) were recorded before, during, and after the seizure. The patients were preoxygenated with a face mask connected to a manual resuscitation device with high-flow oxygen administration and then manually hyperventilated before the stimulus application. The assisted ventilation was followed after the seizure ended until patient emergence from anesthesia.

### 2.3. Clinical and ECT Procedure-Related Variables

Sociodemographic and clinical variables such as age, gender, diagnosis according to DSM-IV-TR criteria, substance use, comorbid medical conditions [39], and the American Society of Anesthesiologists physical status classification system (ASA) [40] were collected from patient medical records. Concomitant medications were categorized using the World Health Organization Anatomical Therapeutic Chemical classification [41] and the defined daily dose (DDD).

During ECT sessions, the following parameters were routinely recorded: ECT parameters (pulse width, energy stimulus intensity, seizure characteristics), frequency of ECT sessions, ASTI (min) [42,43,44,45,46], anesthetic type, anesthetic and muscle relaxant doses, and physiologic variables before, during, and after the session (heart rate, EKG, oxygen saturation, carbon dioxide non-invasively monitored [47,48]).

### 2.4. Evaluation of Seizure Adequacy Parameters

The calculated indexes of quantitative and qualitative aspects of the seizure provided by the Thymatron device regarding amplitude (average seizure energy index, maximum sustained power, time to peak power, early ictal amplitude, midictal amplitude), interhemispheric coherence (maximum sustained coherence, time to peak coherence), degree of abrupt seizure ending and postictal EEG flattening (postictal suppression index), and power spectral analysis were also collected. As these indexes might be altered by artefacts during the recording, the motor and EEG seizure duration plus the postictal suppression rating (PSIr) were visually determined by a psychiatrist with expertise in ECT. The PSIr was analyzed by a single rater using a Likert scale (0–3), with examples and descriptors of the levels of quality [49] based on the Nobler et al. [50] approach.

Given that there is a lack of gold standard indexes or seizure quality scales for evaluating the adequacy of ECT seizures that are universally accepted, two different structured rating scales used in previous research were also assessed to numerically score seizure quality.

The seizure quality index (SQI) awards one point for each accomplished ictal characteristic considered (seizure duration, central inhibition, amplitude, synchronicity, and autonomic activation) according to age-dependent cut-off points. It also classifies the seizure between ideal (>2) and non-ideal [31]. This scoring was used to study the effects of different technical procedures during ECT sessions [11,30,31,33]. Additionally, the SQI has been associated with treatment outcomes, and it was validated in patients undergoing right unilateral brief pulse ECT [23,34].

The seizure adequacy markers sum score (SAMS) takes into account similar markers (seizure length > 25 s, PSI ≥ 80%, wave amplitude ≥ 180 μV, tachycardia ≥ 120 beats per minute, and adequate hemispheric brain wave synchronicity by observation), and based on the score of the fulfilled items, the seizure is considered poor (between 0 and 1.7), medium (1.8–3.4), or good (3.5–5). This scoring showed a significant association with decreases in relapse and depression in patients undergoing brief pulse bitemporal ECT [22].

### 2.5. Statistical Analysis

A multivariate repeated measures regression analysis was performed, as it explicitly takes into account the problem of repeated measurements within an individual [31].

We used the function “lme”/package “nlme” in R package version 3.1-147 [51,52], which allowed us to adjust the effect of mixed linear models, to analyze longitudinal data with multiple measures per subject, and to resolve the non-independence of the measurements. Both the Akaike information criterion (AIC) and Bayesian information criterion (BIC) were used to perform model comparisons. First, we checked whether it was necessary to adjust the models by individual and number of sessions. In the initial model, we controlled the variability of the individual as a random effect, then we added the variability of the session number as a second random effect; subsequently, we also added session number as a fixed term. None of the models that included session number significantly improved the first model that included only the patient as a random effect and that was more parsimonious. Therefore, we used the model that included “Patient” as a random effect as a null model (Null model 0), and we used it as a reference when selecting which factors were regarded as variables of interest and were introduced as fixed terms. The following approach was computed for every dependent variable (SQI [31], SAMS [22], and PSIr [49,50]) to detect which independent factors (sociodemographic, clinical, and procedural variables) predicted each outcome; separately for each factor, we calculated a mixed model that included the factors as fixed terms adjusted by individual as a random effect (univariate analysis). Furthermore, a multivariate model including all the variables that were statistically significant in the univariate models (*p*-value < 0.05) was implemented. From the complete model (which included all significant variables), the model was reduced; the variable that had lowest weight in the model was excluded at each step until there was a final multivariate model where all the variables were significant (backward stepwise process assessed by AIC/BIC). Patient characteristics were described by either mean ± SD or as percentage proportions using IBM SPSS Statistics (IBM Corp. Released 2013. Version 22.0., Armonk, NY, USA).

## 3. Results

### 3.1. Sample Characteristics

The sample included 250 ECT sessions conducted in 47 patients. A minimum of 1 and a maximum of 20 sessions were available per patient (mean 5.3 ± 4.3). The patient characteristics are shown in Table 1.

### 3.2. Outcome Variables

Table 2 shows the analyzed seizure characteristics of the ECT sessions.

Significant results from the univariate repeated measures regression analyses for each of the studied variables are shown in Table 3 and are shown in more detail in Table S1 in the Supplementary Materials (Table S1). Age, ASA classification, hyperventilation, basal oxygen saturation, days between sessions, benzodiazepines, lithium, and tricyclic antidepressants were significant in univariate models as predictors.

All the other variables studied did not show statistically significant results in any of the three seizure quality measures assessed.

The main results of multivariate models, including all the variables that were significant in the univariate models for each of the studied outcomes, are shown in Table 4. The results with the explicative predictors of the final model produced by the backward stepwise regression of the multivariate model are also shown in Table 4 and are shown in more detail in Table S2 in the Supplementary Materials (Table S2).

In the final models, the application of protocolized hyperventilation, the higher oxygen saturation before starting the session, and the more days elapsed between sessions were significant predictors of better seizure quality in both scales used. Regarding the SQI, lower ASA classification was also a significant predictor of seizure quality. On the other hand, benzodiazepine use and lithium daily doses were also predictors of SAMS scores in the final models.

The prediction of the PSIr after the ECT seizure was only significant for the variables succinylcholine dose and stimulus intensity in the univariate and multivariate models. There was an association between higher muscle relaxant doses and lower stimulus intensity applied and the PSIr.

## 4. Discussion

The results of this study show that despite applying the ECT procedure following the clinical guidelines and with anesthetic and stimulus dosages adjusted to provide adequate treatment for each individual, some factors were still related to the obtained seizure quality in the ECT sessions.

Several variables were identified as predictors of seizure quality. Among the patient characteristics, age stood out as a predictor in univariate models, consistent with higher seizure thresholds and weaker seizure expression in elderly individuals [11,30,31,33] despite good clinical outcomes [34]. However, age did not remain significant when studied together with other treatment variables in the multivariate regression models.

ASA classification II more than III was also associated with better scores on both seizure quality scales in univariate analyses and remained a predictor of SQI scores in the final model. These findings suggest worse seizures in patients with medical issues than in healthier patients and with increasing age, although elderly pluripathological patients have good ECT results.

Furthermore, factors related to the procedure that were at least partially modifiable during the treatment were found significant in seizure quality analyses as well.

More days from the last session were associated with better seizure quality results (in all univariate and multivariate models). Similarly, previous research showed that a twice-weekly frequency of ECT was similarly effective as thrice-weekly ECT and may require fewer treatments in acute ECT treatment courses [53].

Ventilation parameters were also found to be associated with seizure quality parameters. Basal oxygen saturation and the application of protocolized hyperventilation were significant in the univariate repeated measures regression analyses, with both remaining significant in the final multivariate models as predictors of both the SQI and the SAMS scores. These findings are consistent with previous research pointing at oxygen, carbon dioxide, and the ratio of concentrations (O_2_/CO_2_) as variables that enhance ECT efficiency through an improvement in seizure length and in other seizure quality parameters [54], such as improved average seizure energy index and time to peak power [55], ictal amplitude and postictal suppression index [56], and seizure quality [31].

Hyperoxygenation prolonged seizure duration [57,58,59,60], and patient basal oxygen saturation levels were correlated with seizure amplitude and postictal suppression [47]. Similarly, correlations were also found between hypocapnia induction (the level of CO_2_ value achieved or the relative decrease below each patient’s baseline values) and some seizure quality indexes [48], although other studies did not find an association between hyperventilation and manually rated seizure amplitude and postictal suppression [36].

Protocolized hyperventilation (asking the patient to voluntarily hyperventilate for a minute plus one minute of facial mask hyperventilation by the anesthesiologist after induction) was previously linked to lower CO_2_ values throughout the entire ECT session and lengthened seizure durations [48,61].

Additionally, it has been described that moderately low PaCO_2_ and high PaO_2_ could act synergistically on seizure quality [31].

Proper ventilation is needed during ECT under anesthetics, and guidelines recommend oxygenation to maintain oxygen saturation near 100% and hyperventilation [61], but is not usually specified how to do it, and standardized ventilation maneuvers are lacking [54]. The ventilation procedures and measures have been poorly defined in studies, and there is heterogeneity among them. Further research is needed on ventilation parameters and their role in seizures [54]. Thus, these results show an easily modifiable factor that could optimize treatment sessions.

Benzodiazepines, lithium, and tricyclic antidepressants were associated with seizure quality indexes in this study, whereas antipsychotics and antiepileptics were not. While psychoactive medication has been considered to impact seizure thresholds, the influence that psychotropics have in ECT has shown inconsistent results, with some authors arguing that they have no effect on seizure adequacy markers, especially when directly compared with other confounders such as stimulation energy, age, and depth of narcosis [11].

Due to their anticonvulsant properties, benzodiazepines and antiepileptics have been the most discussed drugs in the context of ECT, despite their impact on clinical outcomes being less than what would be expected given their pharmacologic effects [62].

The literature regarding the influence of benzodiazepines is contradictory. It has shown the potential of benzodiazepines to shorten seizure duration and decrease treatment efficacy of unilateral ECT [62,63], to compromise the therapeutic effect [64,65], and to have greater effects with high dosages [62]; however in other studies, benzodiazepines had a low impact on seizure quality, only accounting for an influence at high doses [11]. In addition, in some bilateral ECT studies, no detrimental effects on efficacy were observed [12,66], and in another study, high-potency benzodiazepines decreased hemisphere synchronicity and tachycardia, and only the patients who did not receive benzodiazepines achieved good-quality seizures [22].

In the present study, benzodiazepine use had a positive association with seizure quality, agreeing with recent research that found that benzodiazepine use in bitemporal ECT can even improve antidepressant outcomes without occasioning differences in seizure duration or seizure threshold [12].

Antiepileptic treatments were not significant in this study, similar to the literature showing that antiepileptics do not affect EEG quality [22] and that improvements in ECT patients taking antiepileptics are comparable with those who are not taking antiepileptics [10,67].

Regarding the effects of other medications, some previous research did not find an effect of antipsychotics, antidepressants, and lithium on seizure quality [11], while other research found that first-generation antipsychotics improved the quality of hemispheric wave synchronicity, that tricyclic antidepressants improved tachycardia as a seizure quality marker [22], and that lithium potentially prolonged seizures during ECT [11] and reduced the seizure threshold [68].

The PSIr analyses revealed different predictor variables than the seizure quality scales’ predictors. PSI has been used as a standalone adequacy marker correlated with therapeutic outcomes [18], and it was an outcome predictor when controlling for baseline depression rating scores and mode of stimulation [19].

Succinylcholine doses and lower stimulus intensities were associated with better PSIr in this study. We hypothesize that higher succinylcholine doses might diminish electromyography artefacts that may affect PSIr but that might also be associated with younger patients with more muscle mass.

In contrast with previous research that found that the number of ECT sessions, percentage of energy, pulse width, stimulus duration, and frequency were predictors for PSI [7], only the percentage of energy [7,11] among these variables was a predictor in the present study. In previous literature, stimulation energy along with age and high BIS negatively influenced seizure adequacy [11].

PSIr was not associated with ASTI, contrary to the literature that showed that longer ASTI impacted ictal EEG quality indexes, including PSI [36,44]; in addition, there was a study where longer ASTI was linked to superior PSI in the study condition using BIS to determine the moment of seizure induction [35]. However, the mean ASTI observed in this study was similar to the means of long ASTI groups of these studies, so the absence of shorter ASTI and the relative homogeneity of this measure in our sample might explain these differences.

There is a general interest in studying augmentation strategies for overcoming failed seizures during a course of ECT. The study found several modifiable procedural factors that impacted the seizure quality. By adjusting ECT modifiable factors such as ventilation, days elapsed between sessions, stimulus intensity, or muscle relaxant doses, outcomes could be potentially impacted. These factors could be fine-tuned to optimize ECT session results, either at restimulation or in the next session, especially if the obtained SQI score is in the “not ideal” range, if the SAMS score is in the “poor” range, or if there has been a worsening of the ictal EEG.

There are some possible limitations of this study. The population was ethnically homogenous and was based out of a single center. This may limit the generalizability of the findings to other locations and to ECT procedures other than bilateral brief pulses with thiopental or propofol.

Currently, there is no established gold standard for seizure quality evaluation that has been demonstrated to be valid for all ECT modalities. While the SQI—used in this study—has been validated in right-unilateral ECT, it has not been validated in bilateral ECT yet.

The exploratory design of the study involved multiple variables that act together to reflect daily clinical practice rather than involving experimentally controlled scenarios, and we did not correct for multiple testing. The hypotheses generated must be interpreted with caution until they are replicated in future research.

The study shows our daily standard clinical practice where the stimulus doses of ECT sessions were adjusted if needed to counteract the increase in seizure threshold over the treatment course. This fact could mask other possible variables associated with poor seizure adequacy, as practitioners increase the doses to overcome the decrease in seizure parameters. Nevertheless, we identified that, despite proceeding by following the clinical guidelines’ standards and adapting the procedure to provide adequate seizures, several factors still impacted the obtained seizure characteristics, and some of them were modifiable procedural factors that can be optimized to improve ECT session results. Although we did not report outcomes of the complete ECT courses because the aim of the study was the seizure results in each individual session, seizure quality was linked to response [22,23,34] and to decreases in relapse and depression [22] in previous literature.

## 5. Conclusions

The study of seizure adequacy in real-world practice is of utmost relevance as seizure adequacy is prime factor in ECT. Parameters that assure each session’s adequacy help clinicians guide treatment decisions. Therefore, identification of factors that can optimize session quality parameters is necessary for improving treatment.

This study examined the role of clinical and procedure-related variables that influence the results of each ECT session, some of which are modifiable factors. Applying changes to these factors, especially those that have greater influence on the session results, could have a significant impact. In terms of the seizure quality scales, lower age, less severe systemic disease, and concomitant medications (benzodiazepine use and lithium doses) were significant predictors. Higher oxygen saturation before the session, the application of protocolized hyperventilation, and more days elapsed between sessions positively influenced the two scales used. Regarding PSIr, higher muscle relaxant doses and lower applied stimulus intensities were significant predictors. Thus, further studies of different ECT approaches that manipulate these factors during the sessions could identify additional ways of improving ECT.

## Figures and Tables

**Table 1:** Demographic, clinical, treatment, and ECT characteristics of the sample.

Variables	Sample (*N* = 47)
**Demographic characteristics**	
Age, mean (SD)	68.9 (13.3)
<65, *n* (%)	15 (31.9%)
≥65, *n* (%)	32 (68.1%)
Female, *n* (%)	34 (72.3%)
**Medical illnesses**	
Number of non-psychiatric drugs, mean (SD)	3.6 (3.0)
ASA	
ASA II, *n* (%)	25 (54.3%)
ASA III, *n* (%)	21 (45.7%)
**Clinical characteristics**	
*Psychiatric diagnosis*	
Major depressive disorder, *n* (%)	29 (61.7%)
Bipolar disorder, *n* (%)	11 (23.4%)
Schizoaffective disorder, *n* (%)	5 (10.6%)
Schizophrenia, *n* (%)	2 (4.3%)
*Current episode*	
Psychotic symptoms, *n* (%)	25 (53.2%)
Melancholic symptoms, *n* (%)	38 (86.4%)
Catatonic symptoms, *n* (%)	3 (6.5%)
*Pharmacological treatment during ECT*	
Antidepressants, *n* (% yes)	93 (98.9%)
Antipsychotics, *n* (% yes)	66 (70.2%)
Mood stabilizers, *n* (% yes)	11 (11.7%)
Benzodiazepines/Z-drugs, *n* (% yes)	79 (84.0%)
***Characteristics of ECT treatment parameters***	
1-ms pulse width, *n* (%)	20 (42.6%)
0.5-ms pulse width, *n* (%)	27 (57.4%)
Average motor duration across treatments (s), mean (SD)	28.1 (11.3)
Average EEG duration across treatments (s), mean (SD)	43.1 (12.7)

ASA, American Society of Anesthesiologists physical status classification system; ASA II, patients with mild systemic disease; ASA III, patients with severe systemic disease [40]; ms, milliseconds; SD, standard deviation.

**Table 2:** ECT session seizure parameters.

Variables	Sessions Included (*N* = 250)
**Device automated seizure quality indexes**	
Motor seizure duration (s), mean (SD)	25.52 (10.43)
EEG seizure duration (s), mean (SD)	39.96 (11.94)
Average seizure energy index, mean (SD)	9035.20 (11879.7)
Maximum sustained power, mean (SD)	17,025.64 (21,576.28)
Time to peak power, mean (SD)	16.30 (7.96)
Early ictal amplitude, mean (SD)	110.85 (70.26)
Midictal amplitude, mean (SD)	155.61 (90.85)
Maximum sustained coherence, mean (SD)	87.43 (14.39)
Time to peak coherence, mean (SD)	20.83 (11.82)
Delta coherence, mean (SD)	70.76 (24.68)
Postictal suppression index (%), mean (SD)	69.05 (20.36)
**Seizure quality ratings**	
Mean seizure quality sum score (SQI), mean (SD)	2.25 (1.26)
Ideal, *n* (%)	145 (58%)
Non-ideal, *n* (%)	105 (42%)
Mean seizure adequacy markers sum score (SAMS), mean (SD)	2.54 (0.96)
Poor, *n* (%)	25 (10%)
Medium, *n* (%)	179 (71.6%)
Good, *n* (%)	46 (18.4%)
Postictal suppression rating (PSIr ^†^), mean (SD)	1.95 (0.77)
0, *n* (%)	6 (2.4%)
1, *n* (%)	39 (15.6%)
2, *n* (%)	82 (32.8%)
3, *n* (%)	97 (38.8%)
**ECT session parameters**	
Mean ASTI (min), mean (SD)	2.84 (1.36)
Mean stimulus intensity (mC), mean (SD)	325.5 (216.37)
Thiopentone, *n* (%)	141 (96.4%)
Mean thiopentone dose (mg), mean (SD)	178.6 (43.7)
Propofol, *n* (%)	9 (3.6%)
Mean propofol dose (mg), mean (SD)	110.01 (0.5)
Mean succinylcholine dose (mg), mean (SD)	42.0 (12.0)

ASTI, time interval from anesthesia induction to electrical stimulation; mC, millicoulombs; ms, milliseconds; SD, standard deviation. ^†^ [49,50].

**Table 3:** Significant variables in univariate repeated measures regression analysis for the seizure quality index (SQI), the seizure adequacy markers sum (SAMS), and the postictal suppression rating (PSI).

Variables	Seizure Quality Index (SQI)		Seizure Adequacy Markers Sum (SAMS)		Postictal Suppression Rating (PSIr)	
	Estimated	*p*-Value	Estimated	*p*-Value	Estimated	*p*-Value
Protocolized hyperventilation (y/n)	0.362	**0.009**	0.368	**<0.001**	−0.126	0.216
Age (years)	−0.018	**0.049**	−0.019	**0.006**	−0.002	0.638
Basal oxygen saturation (%)	0.119	**0.006**	0.095	**0.003**	−0.041	0.175
ASA (II,III)	−0.471	**0.048**	−0.450	**0.018**	−0.188	0.156
Benzodiazepine use	0.175	0.572	0.262	**0.028**	0.096	0.264
Lithium dose (DDD)	19.676	**0.004**	13.766	**0.012**	0.324	0.430
Tricyclic antidepressant dose (DDD)	0.364	**0.038**	0.327	**0.020**	0.074	0.452
Succinylcholine dose (mg)	−0.004	0.738	−0.005	0.550	0.012	**0.029**
Stimulus intensity (mC)	−0.000	0.762	−0.000	0.641	−0.001	**0.002**
Days from last session	0.018	0.008	*0.012*	**0.027**	−0.002	0.560

AIC, Akaike information criterion; ASA, American Society of Anesthesiologists physical status classification system; ASA II, patients with mild systemic disease; ASA III, patients with severe systemic disease [40]; BIC, Bayesian information criterion; DDD, defined daily dose [41]; mC, millicoulombs; mg, milligrams. The table shows only significant variables. We calculated the mixed model that included the variable of interest as a fixed term adjusting per individual as a random effect. All other evaluated variables were non-significant in the analyses: gender, body mass index (BMI), diagnosis, comorbid medical conditions rated using the validated Spanish version of the Cumulative Illness Rating Scale (CIRS) [39] categories endorsed, severity and total CIRS score, number of drugs for patient somatic illnesses, psychiatric drug categories (antidepressants, antipsychotics, antiepileptics, benzodiazepines/Z-drugs) and daily doses administered of each category, blood pressure, number of treatment sessions, anesthetic type and dosages, time interval from anesthesia induction to electrical stimulation (ASTI), stimulus characteristics (pulse width, stimulus duration, frequency), impedance, motor, and EEG seizure duration. Values in bold indicate statistically significant results.

**Table 4:** Results of multivariate linear mixed effects models of seizure quality associations with clinical and ECT variables.

Variables	Seizure Quality Index (SQI)		Seizure Adequacy Markers Sum (SAMS)		Postictal Suppression Rating (PSIr)	
	Estimated	*p*-Value	Estimated	*p*-Value	Estimated	*p*-Value
*Initial multivariate linear mixed effects model*	Mpm AIC/BIC = 758.0/768.5 Multivariate model AIC/BIC = 743.5/778.3, *p* < 0.001, Marg/R^2^ = 0.172/0.284		Mpm AIC/BIC = 590.2/600.4 Multivariate model AIC/BIC = 566.6/600.4, *p* < 0.001, Marg/R^2^ = 0.251/0.383		Mpm AIC/BIC = 479.8/485.7 Multivariate model AIC/BIC = 469/485.7, *p* < 0.001, Marg/R^2^ = 0.100/0.166	
Protocolized hyperventilation y/n	0.314	**0.025**	0.365	**0.001**	−	*-*
Age (years)	−0.005	0.592	−0.008	0.272	−	*-*
Basal oxygen saturation (%)	0.082	0.057	0.055	0.079	−	*-*
ASA III	−0.384	0.088	−0.331	0.076	−	*-*
Benzodiazepine use y/n	−	−	0.203	0.069	−	*-*
Lithium dose (DDD)	1.096	0.096	0.540	0.362	−	*-*
Tricyclic antidepressant dose (DDD)	0.118	0.446	−0.024	0.868	−	*-*
Succinylcholine dose (mg)	−	−	−	−	0.013	**0.021**
Stimulus intensity (mC)	−	−	−	−	−0.001	**0.005**
Days from last session	0.014	**0.035**	0.006	0.236	−	*-*
*Final multivariate linear mixed effects model: backward stepwise process*	Mpm AIC/BIC = 758.0/768.5 Multivariate model AIC/BIC = 742.3/766.6, *p* < 0.001, Marg/R^2^ = 0.143/0.272		Mpm AIC/BIC = 635.6/646.1 Multivariate model AIC/BIC = 608.4/636.4, *p* < 0.001, Marg/R^2^ = 0.198/0.348		^†^	
Protocolized hyperventilation y/n	0.308	***0.029***	0.351	**0.001**		
Age (years)	−	−	−	−		
Basal oxygen saturation (%)	0.093	**0.030**	0.069	**0.026**		
ASA III	−0.507	**0.018**	−	−		
Benzodiazepine use y/n	−	−	0.322	**0.002**		
Lithium dose (DDD)	−	−	1.237	**0.014**		
Tricyclic antidepressant dose (DDD)	−	−	−	−		
Succinylcholine dose (mg)	−	−	−	−		
Stimulus intensity (mC)	−	−	−	−		
Days from last session	0.018	**0.004**	0.010	**0.040**		

AIC, Akaike information criterion; ASA, American Society of Anesthesiologists physical status classification system; ASA II, patients with mild systemic disease; ASA III, patients with severe systemic disease [40]; BIC, Bayesian information criterion; DDD, defined daily dose [41]; mC, millicoulombs; Mpm: most parsimonious model; mg, milligrams. ^†^ As both variables are significant in the initial multivariate model and this model improves the most parsimonious one, it is not necessary to further reduce this model. Values in bold indicate statistically significant results.

## Data Availability

The datasets generated during the current study are not publicly available because of conditions stipulated by the consent form. However, additional data or analyses are available from the corresponding author upon reasonable request.

## Supplementary Materials

The following are available online at https://www.mdpi.com/article/10.3390/brainsci11060781/s1, Table S1: Detailed results of significant variables in univariate repeated measures regression analysis for the SQI, the SAMS, and the PSI. Table S2: Detailed results of multivariate linear mixed effects models of seizure quality associations with clinical and ECT variables.
