# Supplementary material for: Factors Predicting Ictal Quality in Bilateral Electroconvulsive Therapy Sessions
Source: Brain Sci. 2021 Jun 12;11(6):781. doi: 10.3390/brainsci11060781 (PMC8231613; doi:10.3390/brainsci11060781)
Supplement: Supplementary file 1 [file brainsci-11-00781-s001.zip › brainsci-1218003-supplementary.pdf]

Table S1. Detailed results of significant variables in univariate repeated measures regression analysis for the SQI, the SAMS and the PSI.

Variables	Seizure quality index (SQI)					Seizure adequacy markers sum (SAMS)					Postictal suppression rating (PSIr)				
	**Estimated**	**Standard error**	**Df**	***p-value***	**Marg./Cond. R^2**	**Estimated**	**Standard error**	**Df**	***p-value***	**Marg./Cond. R^2**	**Estimated**	**Standard error**	**Df**	***p-value***	**Marg./Cond. R^2**
Protocolised hyperventilation (y/n)	0.362	0.137	216.382	***0.009***	*0.021/0.266*	0.368	0.098	214.512	***<0.001***	0.038/0.342	-0.126	0.101	203.283	*0.216*	0.006/0.114
Age (years)	-0.018	0.009	46.264	***0.049***	0.033/0.24	-0.019	0.007	45.386	***0.006***	0.069/0.293	-0.002	0.005	43.068	*0.638*	0.002/0.1
Basal oxygen saturation (%)	0.119	0.043	241.573	***0.006***	0.029/0.25	0.095	0.032	238.915	***0.003***	0.031/0.304	-0.041	0.030	219.718	*0.175*	0.008/0.101
ASA (II,III)	-0.471	0.230	38.806	***0.048***	0.036/0.232	-0.450	0.183	40.233	***0.018***	0.056/0.286	-0.188	0.130	41.631	*0.156*	0.014/0.099
Benzodiazepine use	0.175	0.309	42.201	*0.572*	0.003/0.239	0.262	0.118	46.971	***0.028***	0.05/0.309	0.096	0.084	42.6331	*0.264*	0.009/0.104
Lithium dose (DDD)	19.676	0.664	61.703	***0.004***	0.056/0.234	13.766	0.529	57.746	***0.012***	0.048/0.284	0.324	0.407	63.911	*0.430*	0.004/0.107
Tricyclic antidepressant dose (DDD)	0.364	0.171	51.127	***0.038***	0.036/0.254	0.327	0.136	50.939	***0.020***	0.05/0.318	0.074	0.098	51.927	*0.452*	0.004/0.105
Succinylcholine dose (mg)	-0.004	0.010	48.977	*0.738*	0.001/0.238	-0.005	0.008	48.465	*0.550*	0.003/0.299	0.012	0.006	52.264	***0.029***	0.03/0.108
Stimulus intensity (mC)	-0.000	0.001	58.553	*0.762*	0.001/0.281	-0.000	-0.000	0.001	*0.641*	0.002/0.363	-0.001	0.001	51.361	***0.002***	0.067/0.163
Days from last session	0.018	0.007	65.974	*0.008*	0.047/0.237	*0.012*	0.012	0.005	***0.027***	0.036/0.291	-0.002	0.004	73.192	*0.560*	0.002/0.099

AIC, Akaike information criterion; ASA, American Society of Anesthesiologists physical status classification system; ASA II, patients with mild systemic disease; ASA III, patients with severe systemic disease [40]; BIC, Bayesian information criterion; DDD, defined daily dose [41]; df, degrees of freedom; mC, millicoulombs; mg, milligrams; SD, standard deviation.The table shows only significant variables. We calculated the mixed model that included the variable of interest as a fixed term adjusting per individual as a random effect. All other evaluated variables were non-significant in the analyses: gender, body mass index (BMI), diagnosis, comorbid medical conditions rated using the validated Spanish version of the Cumulative Illness Rating Scale (CIRS) [39] categories endorsed, severity and total CIRS score, number of drugs for patient somatic illnesses, psychiatric drug categories (antidepressants, antipsychotics, antiepileptics, benzodiazepines/Z-drugs) and daily doses administered of each category, blood pressure, number of treatment sessions, anaesthetic type and dosages, time interval from anaesthesia induction to electrical stimulation (ASTI), stimulus characteristics (pulse width, stimulus duration, frequency), impedance, motor and EEG seizure duration.

Table S2. Detailed results of multivariate linear mixed effects models of seizure quality associations with clinical and ECT variables.

Variables	Seizure quality index (SQI)					Seizure adequacy markers sum (SAMS)					Postictal suppression rating (PSIr)				
	**Estimated**	**Standard error**	**Df**	**T value**	***p-value***	**Estimated**	**Standard error**	**Df**	**T value**	***p-value***	**Estimated**	**Standard error**	**Df**	**T value**	***p-value***
Initial multivariate linear mixed effects model	Mpm AIC/BIC=758.0/768.5 Multivariate model AIC/BIC=743.5/778.3, p<0.001, Marg/R2=0.172/0.284					Mpm AIC/BIC=590.2/600.4 Multivariate model AIC/BIC=566.6/600.4, p<0.001, Marg/R2=0.251/0.383					Mpm AIC/BIC=479.8/485.7 Multivariate model AIC/BIC=469/485.7, p<0.001, Marg/R2=0.100/0.166				
Protocolised hyperventilation y/n	0.314	0.139	208.195	2.254	***0.025***	0.365	0.103	195.761	3.561	***0.001***	-	-	-	-	-
Age (years)	-0.005	0.009	39.594	-0.541	*0.592*	-0.008	0.007	37.537	-1.116	*0.272*	-	-	-	-	-
Basal oxygen saturation (%)	0.082	0.043	237.169	1.915	*0.057*	0.055	0.031	223.342	1.767	*0.079*	-	-	-	-	-
ASA III	-0.384	0.219	39.879	-1.752	0.088	-0.331	0.182	39.229	-1.823	*0.076*	-	-	-	-	-
Benzodiazepine use y/n	-	-	-	-	-	0.203	0.108	35.997	1.874	*0.069*	-	-	-	-	-
Lithium dose (DDD)	1.096	0.648	54.399	1.693	*0.096*	0.540	0.586	48.941	0.921	*0.362*	-	-	-	-	-
Tricyclic antidepressant dose (DDD)	0.118	0.153	40.446	0.769	*0.446*	-0.024	0.141	32.117	-0.167	*0.868*	-	-	-	-	-
Succinylcholine dose (mg)	-	-	-	-	-	-	-	-	-	-	0.013	0.006	46.484	2.383	**0.021**
Stimulus intensity (mC)	-	-	-	-	-	-	-	-	-	-	-0.001	0.000	52.318	-2.913	**0.005**
Days from last session	0.014	0.006	58.592	2.165	***0.035***	0.006	0.005	68.295	1.195	*0.236*	-	-	-	-	-
Final multivariate linear mixed effects model: backward stepwise process	Mpm AIC/BIC=758.0/768.5 Multivariate model AIC/BIC=742.3/766.6, p<0.001, Marg/R2=0.143/0.272					Mpm AIC/BIC=635.6/646.1 Multivariate model AIC/BIC=608.4/636.4, p<0.001, Marg/R2=0.198/0.348					†				
Protocolised hyperventilation y/n	0.308	0.140	206.372	2.197	***0.029***	0.351	0.100	216.443	3.498	***0.001***					
Age (years)	-	-	-	-	-	-	-	-	-	-					
Basal oxygen saturation (%)	0.093	0.043	237.711	2.178	***0.030***	0.069	0.031	242.848	2.242	***0.026***					
ASA III	-0.507	0.204	38.168	-2.484	***0.018***	-	-	-	-	-					
Benzodiazepine use y/n	-	-	-	-	-	0.322	0.100	46.615	3.229	***0.002***					
Lithium dose (DDD)	-	-	-	-	-	1.237	0.489	63.974	2.520	***0.014***					
Tricyclic antidepressant dose (DDD)	-	-	-	-	-	-	-	-	-	-					
Succinylcholine dose (mg)	-	-	-	-	-	-	-	-	-	-					
Stimulus intensity (mC)	-	-	-	-	-	-	-	-	-	-					
Days from last session	0.018	0.006	58.762	3.001	***0.004***	0.010	0.005	68.342	2.094	***0.040***					

AIC, Akaike information criterion; ASA, American Society of Anesthesiologists physical status classification system; ASA II, patients with mild systemic disease; ASA III, patients with severe systemic disease [40]; BIC, Bayesian information criterion; DDD, defined daily dose [41]; df, degrees of freedom; mC, millicoulombs; Mpm: most parsimonious model; mg, milligrams; SD, standard deviation. † As both variables are significant in the initial multivariate model and this model improves the most parsimonious one, it is not necessary to further reduce this mo
